# Motion-induced position shift in early Alzheimer’s disease

**DOI:** 10.1038/s41598-018-27991-0

**Published:** 2018-06-29

**Authors:** Fei Ye, Maobin Ye, Jun An, Dong Wang, Qin Wang, Yanlin Chen, Xiapei Peng

**Affiliations:** Department of Neurology, The Central Hospital of Wuhan Affiliated to Tongji Medical College, Huazhong University of Science and Technology, Wuhan City, 430014 China

## Abstract

The localization of object position in space is one of the most important visual abilities in humans. Motion-induced position shift is a perceptual illusion in which the position of a moving object is perceived to be shifted in the direction of motion. In this study, we wanted to explore whether and how Alzheimer’s disease (AD) affects this illusion. We recruited a group of patients with early AD and a group of age-matched healthy controls. In our experiments, two drifting Gabor patches moving in opposite directions were presented and participants were asked to report whether the upper Gabor appeared rightwards or leftwards of the lower one. We measured the psychometric functions, of which the point of subjective alignment was taken as the magnitude of motion-induced position shift. We compared the position shift across the two groups at three different retinal eccentricities. We found that position shifts were systematically smaller in the AD group as comparing to the elderly control group. Our data demonstrated that AD patients were less prone to motion-induced position shift. The results add to the existing knowledge of perceptual deficits in AD patients. We suggest that motion induced position shift may be effective as a new behavioral indicator for AD identification.

## Introduction

Alzheimer’s disease (AD) is one of the most prevalent neurodegenerative brain disorders that affect millions of senescent people worldwide^1^. The defining feature of AD is the progressive loss of memory and cognitive functions over the course of disease development^2^. A trend of recent studies reveals that sensory and motor changes may precede the onset of cognitive declines in AD^3^. For instance, it has been shown that early AD is often associated with a variety of visual perceptual deficits^4–7^. In the context of visual motion perception, it is shown that AD patients have elevated coherence thresholds for both simple translational motion^8,9^ and complex optic flow^9–11^, comparing to healthy elderly controls. These motion impairments at the behavioral level are nicely paralleled by recent neurophysiologic studies. Comparing to healthy elderly individuals, AD patients show reduced cortical activations in dorsal visual areas implicated in motion processing^12–15^.

A different line of research has shown that motion processing is not fixed property of the system and it can be biased by many other visual and nonvisual factors^16,17^. In the visual domain, motion perception and position coding in the brain are considered to be intricately linked^18–20^. For example, the perceived position of an object in the environment is not only determined by its retinal input by also modulated by the presence of motion signals in the visual field. Many studies have reported that the position of a moving object appears to be shifted in the direction of motion^20–23^. Such motion-induced position shift suggests the existence of interactions between visual areas specialized for position coding and motion processing. We reasoned that if the cortical motion processing is impaired, e.g., because of AD disease, this may have measurable influence on position perception. So far, it has not been tested whether and how space coding is altered by AD pathology.

In this study, we will address this question by asking whether and how the magnitude of motion-induced position shift is influenced by AD pathology. To this effect, we will conduct psychophysical experiments in a group of AD patients and a group of age-matched normal individuals. We will measure the position shifts and compare them across these two groups of participants. We expect that, relative to normal elderly controls, AD patients will have weakened position shifts due to their impaired motion processing abilities. We will discuss the implications of our study in elucidating the neural mechanisms mediating these perceptual deficits in AD.

## Material and Methods

### Subjects

In this study, we recruited a total of forty volunteers who participated in our experiments. These participants formed into two groups. The first group was made of twenty patients who had early symptoms of AD. The second group consisted of another twenty healthy individuals whose age and sex matched the AD group. Each AD patient was clinically diagnosed according to the National Institute of Neurological and Communicative Disorders and Stroke–Alzheimer’s Disease and Related Disorders Association criteria (NINCDS-ADRDA)^24^. In addition, each AD patient fulfilled the criterion that they had scored above eighteen points but no more than twenty-six based on the Mini Mental State Exam (MMSE score)^25^. The control group was made of normal elderly subjects. Both AD patients and elderly controls had normal or corrected-to-normal vision and were free of eye diseases (e.g., cataract and glaucoma) based on pre-experiment examinations. All participants signed an informed consent for their participation in the current study. The experiments were carried out in full accordance with regulations and guidelines and were approved by the Ethics Committee of Huazhong University of Science and Technology.

### Visual motion stimuli and apparatus

We used the open-source Psychophysics Toolbox to generate the visual motion stimuli in this study^26,27^. The stimuli were shown on a Dell LED monitor with a refresh rate of 100 Hz. The screen resolution was 1024 × 768 pixels. Subjects were instructed to sit in a dimly-lit room with their heads being stabilized by a chin rest. They viewed the monitor binocularly and the viewing distance was set to 57 cm. The visual motion stimuli were a pair of moving Gabor patches displayed on a grey background (30.6 cd/m^2^). Each Gabor was a drifting sinusoidal luminance grating windowed by a Gaussian envelope (SD = 24 arcmin, contrast = 0.5). The grating had a spatial frequency of 1.5 circle/degree drifting at the speed of 10 degree/s.

### Tasks and experimental protocols

**Figure 1:**
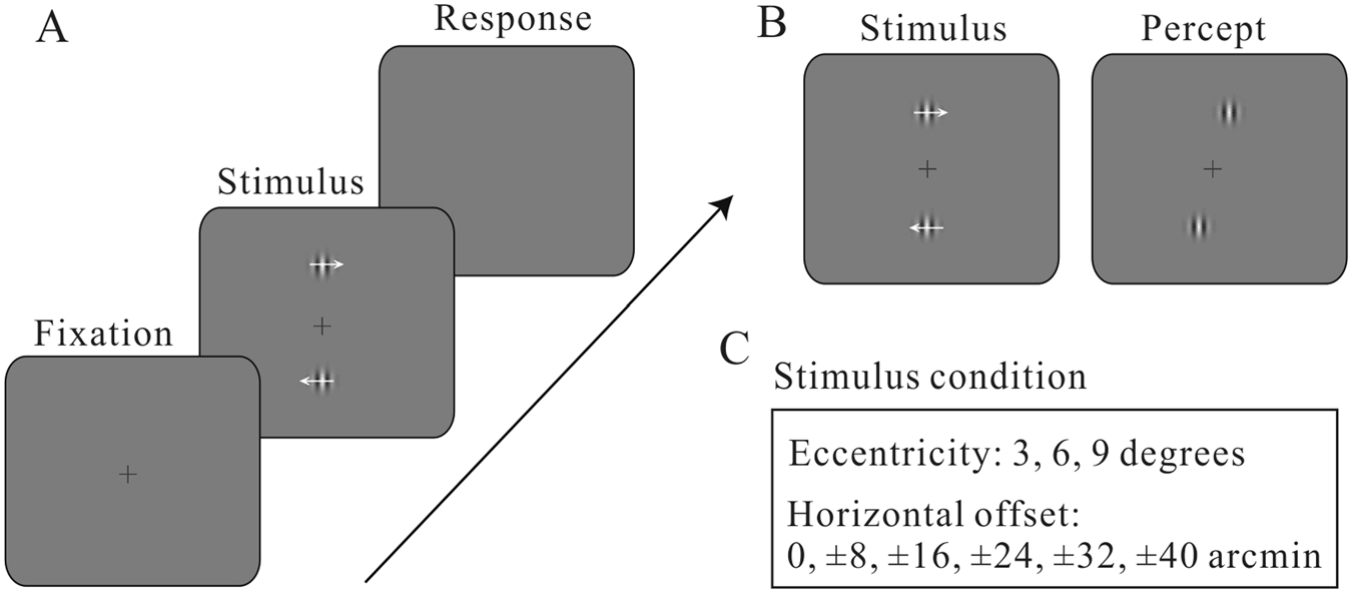
Task designs. (**A**) Observers viewed a pair of drifting Gabor patches moving in opposite directions around fixation (one above and the other one below). They were required to judge whether the upper Gabor is leftwards of the lower one by keyboard presses. (**B**) The schematic illustration of motion-induced position shift. When the two Gabor patches are physically aligned, they appear to be horizontally displaced in the direction of motion. (**C**) Our experiments manipulated the vertical eccentricity of the Gabor patches (relative to the fixation spot) and horizontal offsets between Gabors.

The task flow was illustrated in Fig. 1A. One each trial, subjects were asked to fixate their gazes at the central fixation spot. After a random delay, two Gabor patches were presented simultaneously for 500 ms, one above the fixation spot and the other below, with the same vertical eccentricity relative to the fixation spot (3, 6, or 9 degrees). The upper Gabor was drifting to the right and the lower one drifting in the opposite direction. They had the same drifting velocity. The two Gabors had a variable horizontal offset, which was randomly chosen from a pool of eleven values (0, ±8, ±16, ±24, ±32, ±40 arcmin). Positive values mean rightward offsets (upper Gabor is to the right of the lower one) and negative ones indicate leftward offsets. After stimulus presentation, subjects were required to judge the relative horizontal position of the two Gabors, and report by keyboard presses whether the top stimulus appeared leftwards of the bottom one.

We used the method of constant stimuli to measure the psychometric performance of each subject. The task conditions (3 vertical eccentricities, 11 horizontal offsets) were randomized on a trial-by-trial basis. Each task condition was performed 15 repetitions. Before data collection, each subject was given a few practice trials to familiarize themselves with the task. During the experiment, we allowed subjects to pause the task whenever they need a break.

### Data analysis and statistics

Data analysis were conducted in MatLab (MathWorks, R2015a). For each subject, we first computed the psychometric performance based on their responses. We then fitted the data with a cumulative Gaussian function. The point of 50% performance corresponded to the point of subjective alignment (PSA) between the two moving Gabors and was taken as the magnitude of motion-induced position shift. PSA was extracted at each retinal eccentricity from each subject. These PSAs were then averaged across populations within each participant group at each retinal eccentricity. We conducted two statistical analysis on the populated PSA data. Firstly, we performed a mixed two-way ANOVA on the PSA (within-subject factor: retinal eccentricity; between-subject factor: subject group). Secondly, we conducted post-doc unpaired *t*-tests between groups at each eccentricity. For all statistical analysis the level of significance was set at p < 0.05.

## Results

**Figure 2:**
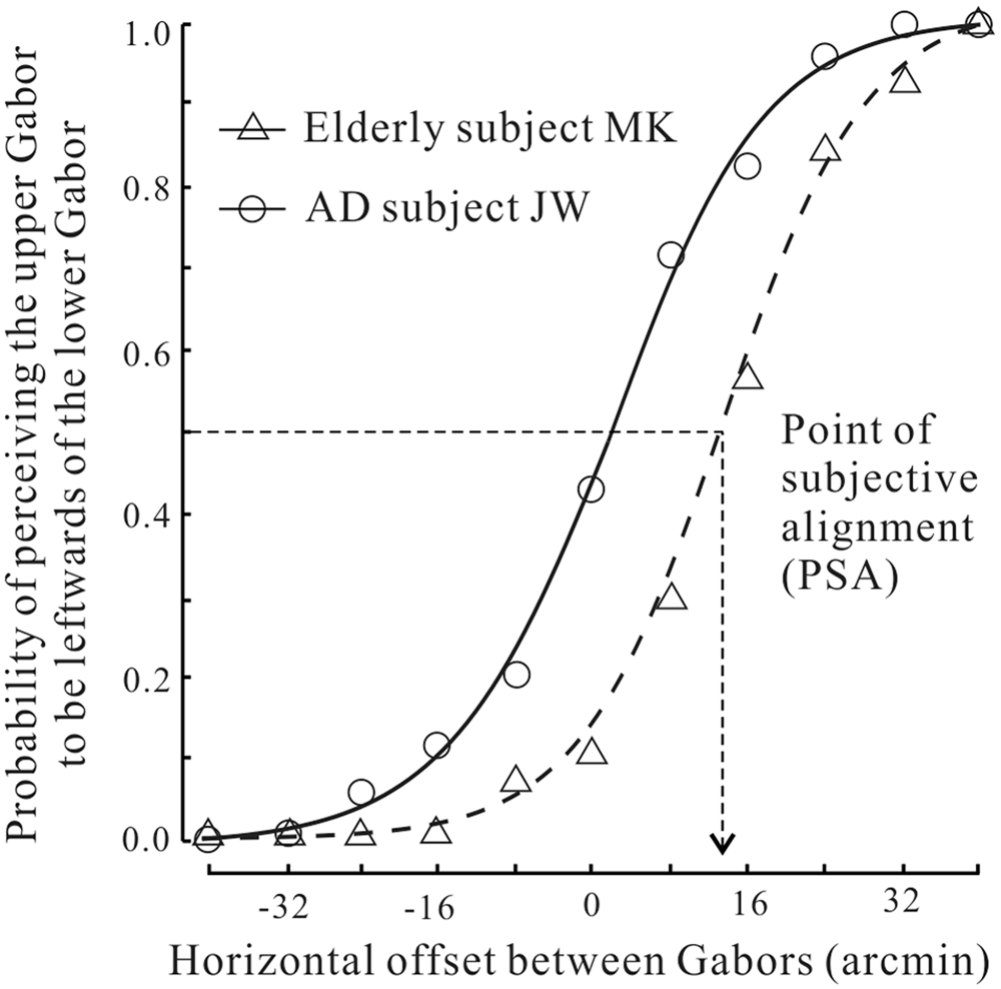
The psychometric measurement of position shifts in two representative subjects. The y-axis plotted the probability of perceiving the upper Gabor to be leftwards of the lower one as a function of horizontal offset. Data were shown for one AD patient and one elderly subject at 3° eccentricity. We fitted a cumulative Gaussian function to the data and the point of 50% performance was taken as the magnitude of position shift.

For each participant at each retinal eccentricity, we calculated the probability of reporting the upper Gabor patch to be leftwards of the lower one as a function of horizontal offset. Figure 2 shows the psychometric functions for two representative participants at the same eccentricity of 3°. Open circles refer to the data from the AD patient JW, and open triangles the data from the elderly subject MK. For the healthy elderly subject MK, the perceived locations of Gabor patches were greatly displaced in their drifting directions. This was consistent with findings from many previous studies^21,22^. Specifically, the upper Gabor drifting rightwards appeared to be shifted to the right relative to its physical location, and the opposite was true for the lower Gabor. As a result, the probability of seeing the upper Gabor as more leftwards than the lower one was systematically reduced than expected based on their physical offsets. For example, at the 0 arcmin offset in which the two Gabors were physically aligned (the probability should be 50%), the probability in perception was around 10%. This means that the physically aligned Gabors appeared to be misaligned in the direction of motion. To achieve the perceptual alignment, the top stimulus needed to be physically dislocated 14.2 arcmin to the left of the bottom one. In contrast, for the AD patient JW, the probability at the 0 arcmin offset was close to 50%, and it took only 1.7 arcmin to appear aligned. In other words, motion leads to large perceptual shift in the healthy control subject and small shift in the patient subject.

**Figure 3:**
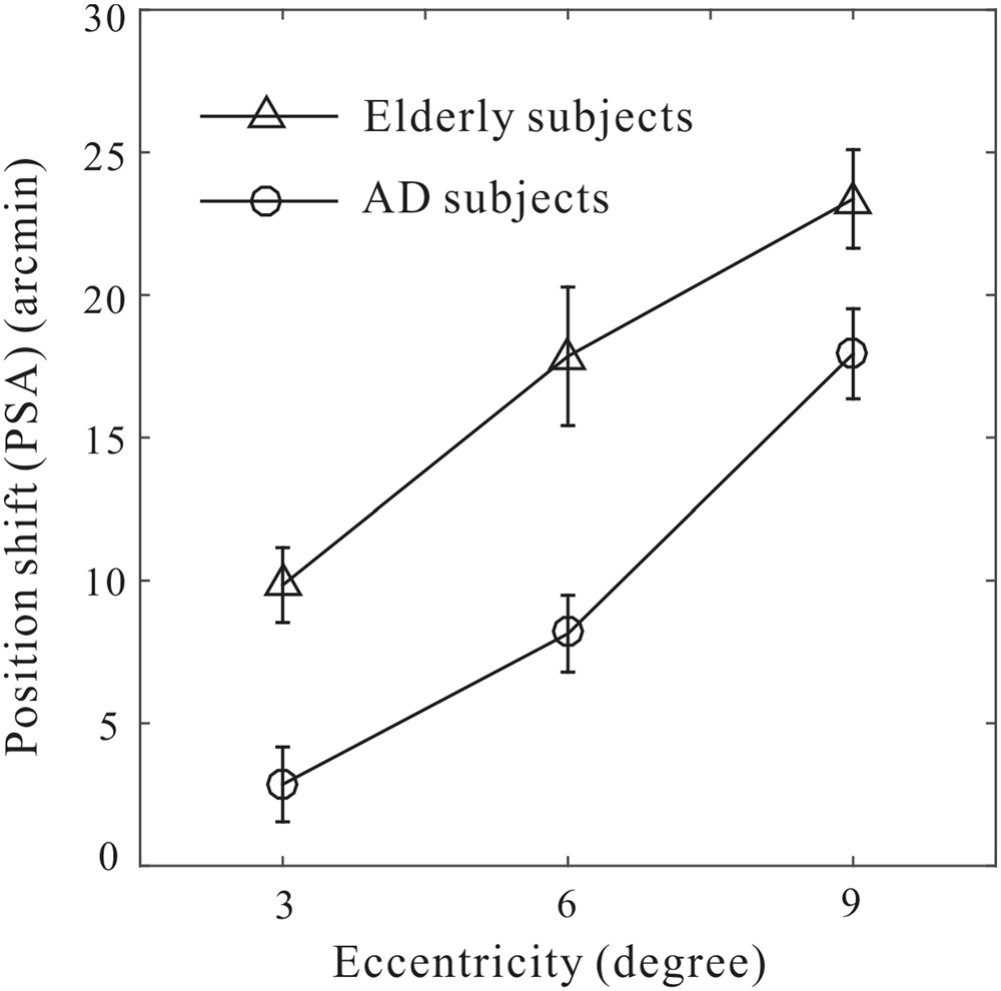
The dependence of position shift on the retinal eccentricity in two groups of participants. Results are shown for mean ± SEM averaged within each group.

To quantitively assess these motion-induced position shifts and compare them between the two groups at different retinal eccentricities, we fitted a cumulative Gaussian function to the raw psychometric data for each participant and at each retinal eccentricity. We then extracted the point of subject alignment (PSA, 50% performance) as the magnitude of motion-induced position shift. Figure 3 plotted the averaged position shifts (PSA data) against three retinal eccentricities for the AD and elderly groups respectively. Two observations were noteworthy. Firstly, the magnitude of motion-induced position shift increased with increasing retinal eccentricity, and this was true for both the elderly and AD groups. Secondly, position shifts were much weaker in the AD group than those in the elderly control group. Statistically, we performed a two-way mixed-design ANOVA analysis. The analysis revealed that there were significant main effects on the factor Group (F(1,38) = 27.3, p < 0.001)) and the factor Eccentricity (F(2,76) = 38.5, p < 0.001)), but no significant interactions between the two factors (F(2,76) = 0.88, p = 0.42). In addition, a post-doc unpaired *t*-test between groups showed that the differences in the magnitude of position shifts were significant at each retinal eccentricity (all p < 0.05).

## Discussions

The position of a moving object appears to be displaced in the direction of motion. The current study exploited this well-established phenomenon and examined the properties of position shift in patients with early AD. We found that position shifts were systematically weakened in AD patients than in healthy observers. Biasing the perceive location of a moving object in the direction of movement is an important visual function, as it allows the observers to compensate for neural delays in the visual processing stream to accurately interact with the moving object^18^. Our results of reduced position shift in AD group indicates that the onset of AD pathology not only impairs motion processing, as reported in several previous studies^8,9^, but also alters position coding in the brain. Our results suggest that motion-induced perceptual mis-localization may be used as a new behavioral marker for the identification and detection of AD pathology in aging populations.

What does the impaired position shift at the behavioral level inform us about the cortical mechanisms underlying AD pathology? Or the alternative question we could ask is why motion-induced position shift is weakened in AD patients? To address these questions, we need to review the literature on the neural correlate of motion-induced position shift. Spatial coding relies on the properties of primary visual cortex (V1) which contains neurons with spatially-localized receptive fields (RF)^28^, while motion information is coded by a cascade of neural networks including lower-level V1 and middle temporal area (MT)^29,30^. Thus, the effect of motion-induced position shift is generally explained by two different accounts. The first account attributes the position shift to early visual cortex V1. This account is supported by single-unit studies showing that motion displaces the RFs of cat V1 neurons^31^ and human fMRI studies showing that motion-induced position shift activates primarily early visual cortex^32^. The second account holds that the position shift is mediated by top-down modulations from higher motion-sensitive area MT to lower area V1 which then biases the position coding in V1. This account has also been supported by evidences from recent studies revealing motion-dependent neural representation of object position in human MT+^33,34^ and many psychophysical experiments reporting that motion-induced position shift occurs after motion integration which presumably activates higher visual area such as MT^35–37^.

The first account implies that AD pathology may be associated with the changes in the RF properties of V1 neurons. Specifically, the RFs of V1 neurons in AD patients might be shifted less in the presence of motion, leading to attenuated position illusion in perception. Whether this is true remains to be tested, although it is ethically difficult to access the responses of single neuron in human subjects. According to the second account of motion-induced position shift, there are two possible explanations for AD. The first possibility is that the weakened position shift is attributable to motion processing deficits in higher area MT of AD patients. This interpretation is compatible with findings in the AD literature. More specifically, previous studies have shown that AD patients are less sensitive to motion signals as they had elevated coherence thresholds relative to healthy controls^8,9^ and that AD attenuates the cortical responses of higher level motion-sensitive areas^12,14,15^. These perturbations in higher areas could then lead to weakened influence on the position coding of V1 neurons. The alternative possibility is that the connectivity efficacy from MT to V1 might be undermined and this could cause reduced position shift in V1 coding even though MT response is preserved in AD pathology. This interpretation resonances with the notion that several neurological disorders including AD may be associated with impaired network connectivity in the brain^13,38–41^. Nevertheless, we should bear in mind that, these two possibilities are not mutually exclusively, as they are referring to different properties of the network. It is likely that reduced MT response and disrupted MT-V1 connectivity might just co-exist in patients with AD pathology. Future studies with more elaborate designed are needed to further tease them apart.

We note that motion-induced position shift is affected not only by retinal eccentricity but also by a number of other stimulus parameters such as the spatial and temporal aspects of the motion signal^42^. In the current study, we varied only the retinal eccentricity because we were limited by the number of trials the senior and AD participants could perform in our experiments. Further studies are needed to validate and fully characterize the properties of position shift reduction in AD under a variety of motion parameters. Another limitation of the current study is that we sampled only early AD patients. It remains to be explored whether and how such effects apply to patients with more severe symptoms. Nevertheless, the current study has important implications on the AD research. Position shift might provide an alternative measure of visual dysfunction in AD and thus may be useful in the future detection and characterization of AD pathology before or in junction with the traditional clinical evaluations.
